# Pulmonary gas exchange and acid–base status during immobilisation of black rhinoceroses (*Diceros bicornis*) in Zimbabwe

**DOI:** 10.4102/jsava.v87i1.1328

**Published:** 2016-12-02

**Authors:** Åsa Fahlman, Anna Edner, Sandra Wenger, Chris Foggin, Görel Nyman

**Affiliations:** 1 Department of Clinical Sciences, Swedish University of Agricultural Sciences, Sweden; 2 Clinic for Zoo Animals, Exotic Pets and Wildlife, Vetsuisse Faculty, University of Zürich, Switzerland; 3 Victoria Falls Wildlife Trust, Victoria Falls, Zimbabwe

## Abstract

When immobilising wildlife, adverse side effects can include hypoxaemia, acidosis and hypertension. Pulmonary gas exchange and acid–base status were evaluated during immobilisation of 25 free-ranging and one boma-held black rhinoceros (*Diceros bicornis*) in Zimbabwe. The effect of different body positions on arterial oxygenation was evaluated. A combination of the following drugs was used: an opioid (etorphine or thiafentanil), azaperone and an α_2_-adrenoceptor agonist (detomidine or xylazine). Respiratory and heart rates, rectal temperature and pulse oximetry–derived haemoglobin oxygen saturation were recorded. Serial arterial blood samples were analysed immediately in the field. Marked hypoxaemia and hypercapnia were recorded in immobilised free-ranging black rhinoceroses. Arterial oxygenation was higher during sternal compared to lateral recumbency. Most rhinoceroses developed acidaemia of respiratory and metabolic origin. Initially high lactate concentrations in free-ranging rhinoceroses decreased during immobilisation. Pulse oximetry was unreliable in the detection of hypoxaemia. Positioning in sternal recumbency and routine use of oxygen supplementation are recommended in the management of immobilised rhinoceroses as measures to improve arterial oxygenation.

## Introduction

Immobilisation of free-ranging rhinoceroses in southern Africa is often carried out as an important part of management and conservation of both black rhinoceroses (*Diceros bicornis*) and white rhinoceroses (*Ceratotherium simum*). When immobilising wildlife, it is our responsibility to ensure optimal handling to minimise capture-related morbidity and mortality. This is extra critical for endangered species, where each individual may be essential for survival of the species.

The potent opioid etorphine that is commonly used for immobilisation of rhinoceroses causes respiratory depression (Haigh 1990). Adverse effects in immobilised rhinoceroses include hypoxaemia, acidosis and hypertension (Bush *et al*. 2004; Hattingh, Knox & Raath 1994; Heard, Olsen & Strover 1992). Severe hypoxaemia and acidosis can predispose animals to cardiac arrhythmias, organ failure, capture myopathy and death (Spraker 1993). Pulmonary function during anaesthesia is impaired by body positions such as lateral or dorsal recumbency (Gleed & Dobson 1988), with a higher risk of hypoxaemia in heavy, round-bellied animals, as shown in horses (Moens 1989). Recommended positioning of rhinoceroses during immobilisation has varied between sternal and lateral recumbency (Kock *et al*. 1995; Morkel *et al*. 2010; Radcliffe & Morkel 2014; Wenger *et al*. 2007). A recent study demonstrated that black rhinoceroses immobilised with etorphine and azaperone experienced more severe hypoxaemia and lactic acidaemia during lateral compared to sternal recumbency (Radcliffe *et al*. 2014).

A rapid induction is important to reduce the risk of injury, exertion and hyperthermia when immobilising free-ranging rhinoceroses in rough terrain or during hot conditions. The fentanyl derivative, thiafentanil, is rapid-acting and produces quick induction times in different wildlife species (Janssen *et al*. 1993; Stanley *et al*. 1988), but has not been evaluated in black rhinoceroses, which often inhabit thick bush in mountainous terrain.

Arterial blood gas values during immobilisation with different drug combinations have been reported for both captive and free-ranging white rhinoceroses (Boardman *et al*. 2014; Bush *et al*. 2004; Cornick-Seahorn *et al*. 1995; Hattingh *et al*. 1994; Haw *et al*. 2014; Heard *et al*. 1992; Kock & Pearce 1985; Miller *et al*. 2013; Walzer *et al*. 2000). In black rhinoceroses, arterial blood gases have been described in a captive individual anesthetised with isoflurane (Ball *et al*. 2001), and in free-ranging black rhinoceroses immobilised with etorphine and azaperone (Radcliffe *et al*. 2014). The principal aim of this study was to evaluate pulmonary gas exchange and acid–base status in black rhinoceroses immobilised with drug combinations including an opioid, azaperone and an α_2_-adrenoceptor agonist. In addition, the effect of different body positions on arterial oxygenation was evaluated.

## Materials and methods

### Animals and study areas

The study included 25 free-ranging and one boma-held black rhinoceros. The reasons for immobilisation included ear notching, health examination, snare removal and translocation. Immobilisations were carried out in the Malilangwe Wildlife Reserve and the Bubiana, Chiredzi River, and Save Valley Conservancies in Zimbabwe between 2002 and 2007. Elevation ranged from 350 m a.s.l. to 500 m a.s.l.; barometric pressure was approximately 730 mmHg.

### Capture methods, drugs and darting equipment

Free-ranging rhinoceroses were located using trackers and a fixed-wing aircraft. Once located, the animals were darted in the hindquarters from a helicopter with a drug combination of an opioid (etorphine or thiafentanil), azaperone and, in all but one animal, an α_2_-adrenoceptor agonist (detomidine or xylazine). For darting, 3-mL Cap-Chur^®^ syringes with 4.5 cm – 6.0 cm bevelled, barbed needles (Palmer Cap-Chur Inc., Powder Springs, Georgia, USA) and Simmons tailpieces were fired from a powder charge rifle (Pneu-Dart Inc., Williamsport, Pennsylvania, USA). Hyaluronidase (lyophilised powder, 5000 IU/vial; Kyron Laboratories [Pty] Ltd., Benrose, South Africa) was added at 1250 IU – 8000 IU per dart to the free-ranging rhinoceroses.

The boma-held black rhinoceros (subadult male) was immobilised three times because of a snare injury. It was darted in the muscles of the neck with 1.7 mg etorphine and 30 mg – 40 mg azaperone, using 3-mL Pneudart^®^ syringes with 3.8-cm needles (Pneu-Dart Inc.).

Once the free-ranging rhinoceroses were recumbent, nalorphine (Nalorphine^®^, 20 mg nalorphine hydrobromide/mL; Kyron Laboratories [Pty] Ltd) was administered intravenously (i.v.). Thereafter, if pulse oximetry readings of the haemoglobin oxygen saturation (SpO_2_) were below 85%, incremental doses of nalorphine were administered at the discretion of the veterinarian in charge. For reversal of immobilisation in the field or at the boma, diprenorphine was administered i.v. at 2.2–3.5 times the etorphine dose or naltrexone intramuscularly at 20 times the thiafentanil dose.

### Monitoring

The time from darting until recumbency (induction time) and from darting until reversal of the immobilisation (total immobilisation time) were recorded. The distance the free-ranging rhinoceros moved after sighted from the helicopter until recumbency was subjectively estimated visually by the helicopter crew. Once recumbent, a blindfold and earplugs were placed to reduce external stimuli. The rhinoceroses were positioned in sternal recumbency when possible. Physiological variables were monitored throughout the procedure and recorded every 5–10 min. Respiratory rate was monitored by observing chest movements or by counting the exhalation of air from the nostrils. Rectal temperature was monitored with a digital thermometer with continuous reading and a measurement range of 28.9 °C – 42.2 °C (Welch Allyn Diatec 600; Welch Allyn, Inc., Skaneateles Falls, New York, USA). Outdoor temperature ranged from 20 °C to 36 °C. It was measured with a thermometer that was placed in the shade near the rhinoceros. During hot days or if the rectal temperature reached 39.0 °C, the rhinoceros was cooled with water. Haemoglobin oxygen saturation (SpO_2_) was monitored continuously by pulse oximetry (Nellcor NPB-40 or Nellcor N-20 Handheld Pulse Oximeter [Nellcor Inc., Pleasanton, California, USA] or Tuffsat^®^ Pulse Oximeter [Datex-Ohmeda Inc., Madison, Wisconsin, USA]). The pulse oximeter probe was placed on the ear after scraping the skin on both sides of the ear with a scalpel blade until the cartilage was seen. Heart rate was monitored by pulse oximetry or by auscultation.

### Blood sampling and analysis

Single or serial arterial blood samples were collected for blood gas and acid–base analysis. The samples were collected anaerobically from an auricular artery on the medial aspect of the ear using 2-mL pre-heparinised syringes and 21-gauge needles.

All samples (whole blood) were processed immediately in the field using a portable analyser (i-STAT^®^1 Portable Clinical Analyser; Abbott Laboratories, Abbott Park, Illinois, USA). The analysis included measured values for pH, lactate and partial pressures of carbon dioxide (PaCO_2_) and oxygen (PaO_2_), whereas arterial haemoglobin oxygen saturation (SaO_2_), actual base excess (BE) and actual bicarbonate (HCO_3_) were calculated. Blood gas values and pH were corrected to the rectal temperature. Hypoxaemia was defined as mild (PaO_2_ 8.0 kPa – 10.7 kPa [60 mmHg – 80 mmHg]) or marked (PaO_2_ < 8.0 kPa [< 60 mmHg]). Acidaemia was defined as a pH < 7.35 and hypercapnia as a PaCO_2_ > 6.0 kPa (45 mmHg), which was marked if PaCO_2_ was > 8.0 kPa (60 mmHg).

The alveolar–arterial oxygen tension difference [P(A–a)O_2_] at standard temperature (37 °C) was estimated by calculation according to the equation:

$${\text{PAO}}_{2}={\text{F}}_{1}{\text{O}}_{2}({\text{P}}_{\text{B}}-{\text{P}}_{\text{H}2\text{O}})-({\text{PaCO}}_{2}/\text{RQ}),$$ [Eqn 1]

where PAO_2_ = partial pressure of alveolar oxygen; F_I_O_2_ = fraction of inspired oxygen (0.21); P_B_ = barometric pressure; P_H2O_ = saturated vapour pressure for water at 37 °C (6.3 kPa [47 mmHg]) and RQ = respiratory quotient (assumed to be 1.0, primarily carbohydrate metabolism).

To describe the contribution of different factors that influenced the arterial oxygenation (PaO_2_), the difference between the estimated PAO_2_ if awake at sea level (optimal PAO_2_) and the actual measured PaO_2_ during anaesthesia at the study area was considered 100%. The relative contribution of the following factors was calculated for altitude = the difference between estimated PAO_2_ awake at sea level (optimal PAO_2_) and estimated PAO_2_ awake at the study area; hypoventilation = the difference between estimated PAO_2_ awake at the study area and the estimated PAO_2_ anaesthetised at the study area; intrapulmonary factors = the difference between the estimated PAO_2_ anaesthetised at the study area and the actual PaO_2_ anaesthetised at the study area (Fahlman *et al*. 2011). A detailed description on the above calculations is presented elsewhere (Fahlman *et al*. 2011).

### Data analysis

The change per minute in blood gases, pH (37 °C) and lactate was calculated with a one-sample *t*-test. Difference in PaO_2_ between body positions (sternal versus lateral recumbency) and age groups (adults, subadults and calves) at approximately 15 min after darting were analysed with a two-way main-effects analysis of variance. Statistical analyses were done with Procedure Mixed, SAS^®^ System 9.1 (SAS Institute Inc., Cary, North Carolina, USA). A *p* < 0.05 was considered significant in all analyses.

## Results

Etorphine was used in combination with azaperone in 1 animal, with azaperone and xylazine in 18 animals and with azaperone and detomidine in 4 animals (Table 1). Thiafentanil was used with azaperone and detomidine in one adult and with azaperone and xylazine in one subadult (Table 1). Recumbency occurred within 2–10 min (mean 5 min) of darting in free-ranging black rhinoceroses immobilised with etorphine combinations. The induction times for the two rhinoceroses immobilised with thiafentanil combinations were 4 and 5 min, respectively. The estimated distance the animals moved after being sighted from the helicopter until recumbency was 0.3 km – 4.9 km (mean 1.6 km). Total immobilisation time ranged between 36 and 164 min (mean 68 min) in free-ranging rhinoceroses.

**TABLE 1a:** Immobilising drug combinations and doses used for free-ranging black rhinoceros in Zimbabwe. Doses (in mg) for black rhinoceros immobilised with etorphine, azaperone, and xylazine *or* detomidine.

Age group†	*n*	Sex‡	Etorphine§	Azaperone¶	Xylazine††	Detomidine‡‡
Adult	16	11 M, 5 F	3.2–4.3	70–90	20–45 (*n* = 14)	3.5–4.0 (*n* = 2)
Subadult	2	2 M	2.7–2.8	35–40	20 (*n* = 1)	2.5 (*n* = 1)
Calf§§	5	3 M, 2F	1.0–1.7	15–22	10–13 (*n* = 3)	1.0 (*n* = 1)

† , Age group definitions: adult > 4 years, subadult 2–4 years, calf 1–2 years;

‡ , M, male; F, female;

§ , M99^®^, 9.8 mg etorphine HCl/mL, Novartis South Africa (Pty) Ltd., Kempton Park, South Africa;

¶ , Stresnil^®^, 40 mg azaperone/mL, Janssen Animal Health, Halfway House, South Africa;

†† , Rompun^®^, 500 mg xylazine powder/vial, Bayer, Leverkusen, Germany;

‡‡ , Domosedan^®^, 10 mg detomidine HCl/mL, Novartis South Africa (Pty) Ltd.;

§§ , one calf did not receive an alpha-2-adrenoceptor agonist.

**TABLE 1b:** Immobilising drug combinations and doses used for free-ranging black rhinoceros in Zimbabwe. Doses (in mg) for black rhinoceros immobilised with thiafentanil, azaperone, and xylazine *or* detomidine.

Age group†	*n*	Sex‡	Thiafentanil§	Azaperone¶	Xylazine††	Detomidine‡‡
Adult	1	1 M	3.0	90	-	4.5
Subadult	1	1 F	2.0	50	20	-

† , Age group definitions: adult > 4 years, subadult 2–4 years, calf 1–2 years;

‡ , M, male; F, female;

§ , A3080, 10 mg thiafentanil oxalate/mL, Wildlife Pharmaceuticals Inc., Fort Collins, Colorado, USA;

¶ , Stresnil^®^, 40 mg azaperone/mL, Janssen Animal Health, Halfway House, South Africa;

†† , Rompun^®^, 500 mg xylazine powder/vial, Bayer, Leverkusen, Germany;

‡‡ , Domosedan^®^, 10 mg detomidine HCl/mL, Novartis South Africa (Pty) Ltd.

**FIGURE 1:**
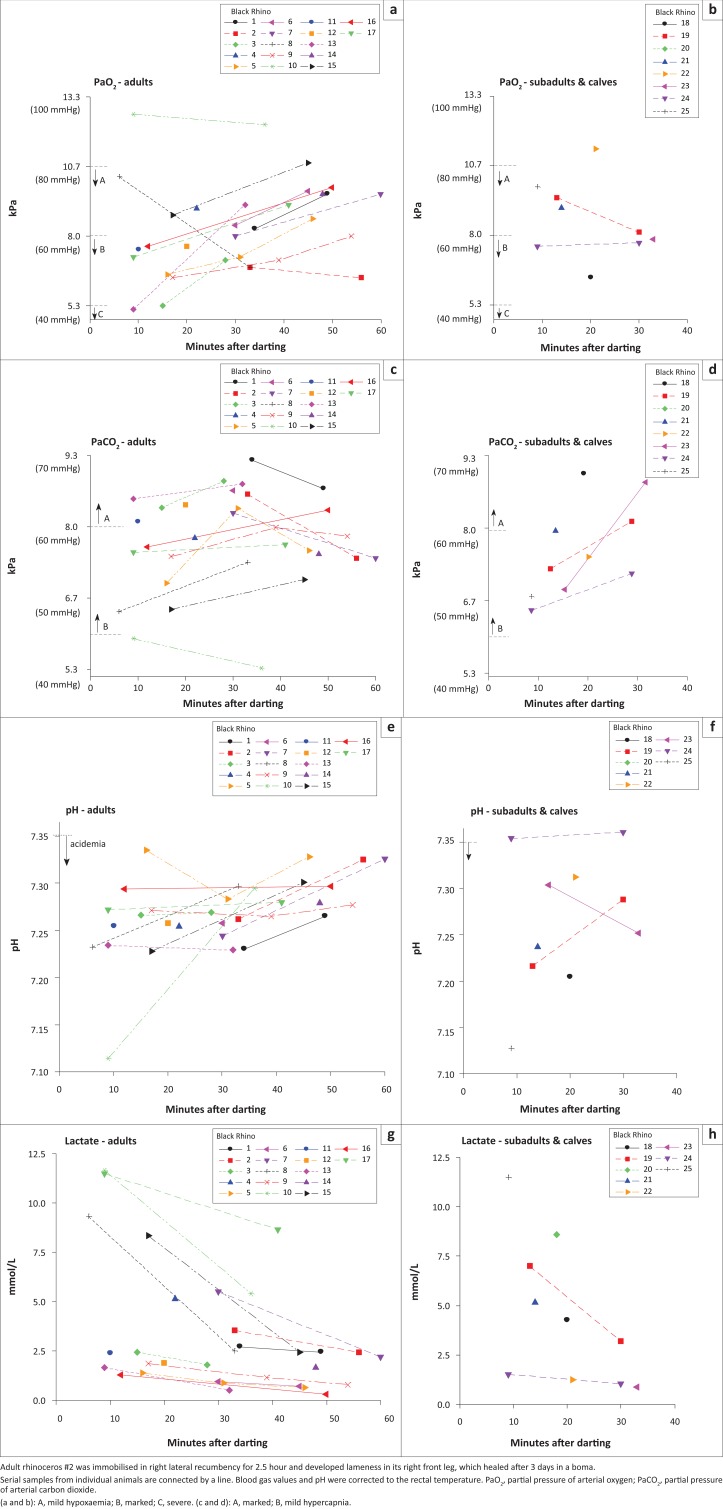
(a–h), Arterial blood gases, pH and lactate concentration in 25 free-ranging black rhinoceroses immobilised with a combination of an opioid (thiafentanil in adult #3 and subadult #18, etorphine in the others), azaperone and an α_2_-agonist (xylazine or detomidine).

One to three arterial samples were collected from each rhinoceros 6–76 min after darting, depending on the ongoing procedure. From all but six rhinoceroses, the first sample was collected after nalorphine had been administered for partial reversal of the opioid effect. Total doses of incremental nalorphine administered ranged between 1 mg and 8 mg. Physiological data from free-ranging black rhinoceroses are presented descriptively in Figure 1. Most free-ranging rhinoceroses developed hypoxaemia, hypercapnia and acidaemia. The PaO_2_ and SaO_2_ increased (*p* = 0.025 and *p* = 0.018, respectively) and lactate decreased (*p* = 0.001) significantly over time. There was no significant change over time in pH, PaCO_2_ and SpO_2_. Initially, of the 25 free-ranging rhinoceroses, hypoxaemia was recorded in 23 individuals (range of PaO_2_ 5.2 kPa – 10.3 kPa, median 7.9 kPa [range 39 mmHg – 77 mmHg, median 59 mmHg]) and 13 of the 23 had marked hypoxaemia. Eleven of the 13 rhinoceroses with a marked hypoxaemia had received nalorphine. In the two rhinoceroses that were immobilised with thiafentanil combinations, some of the lowest oxygen tensions were measured (PaO_2_ 5.3 kPa and 6.4 kPa, respectively [40 mmHg and 48 mmHg]).

**FIGURE 2:**
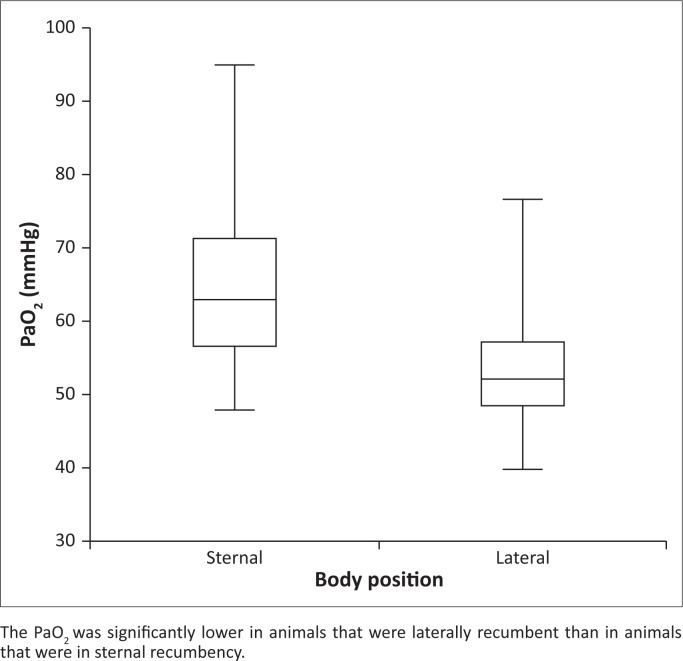
Box-plot showing partial pressure of arterial oxygen (PaO_2_) (median, 25th and 75th quartiles, range) in 21 free-ranging black rhinoceroses during sternal (*n* = 11) or lateral (*n* = 10) recumbency at approximately 15 minutes after darting.

The values of haemoglobin oxygen saturation measured by pulse oximetry (SpO_2_) were higher than the values derived from the i-STAT^®^1 (SaO_2_) in 34 of 47 paired measurements. In 18 of 34 measurements, the difference was over 5 percentage points. In free-ranging rhinoceroses (all age groups), the PaO_2_ values were significantly higher during sternal compared to lateral recumbency (Figure 2).

The P(A–a)O_2_ was > 10 mmHg in all rhinoceroses and > 20 mmHg in all but two. In free-ranging adult rhinoceroses, the mean ± s.d. values for P(A-a)O_2_ were 44 mmHg ± 7 mmHg during lateral recumbency and 29 mmHg ± 9 mmHg during sternal recumbency, approximately 15 min after darting. Based on calculations including estimated PAO_2_ values and measured PaO_2_ values, altitude (barometric pressure) was responsible for approximately 15% of the reduction in the mean value of PaO_2_, hypoventilation contributed approximately 10% and intrapulmonary factors accounted to approximately 75%.

In the boma-held rhinoceros, which was immobilised on three different occasions, the PaO_2_ ranged between 8.1 kPa and 11.1 kPa (61 mmHg – 83 mmHg) and the PaCO_2_ ranged between 6.5 kPa and 7.3 kPa (49 mmHg – 55 mmHg).

In all but one black rhinoceros, acidaemia (pH 7.11–7.34) and hypercapnia (PaCO_2_ 6.4 kPa – 9.2 kPa [48 mmHg – 69 mmHg]) were recorded, and in 13 free-ranging animals the hypercapnia was marked (Figure 1). Two free-ranging rhinoceroses had a pH < 7.2 and lactate concentrations > 11 mmol/L. The highest lactate concentration measured after helicopter darting was 11.7 mmol/L (Figure 1) compared to 3.4 mmol/L after darting in the boma. In all rhinoceroses, lactate concentration decreased over time, whereas BE (range -15 mmol/L – 5 mmol/L) and HCO_3_ (range 14 mmol/L – 30 mmol/L) increased.

The rectal temperature ranged from 37.7 °C to 40.1 °C at the times of arterial sampling. Rectal temperatures > 39.0 °C were recorded in 11 free-ranging rhinoceroses, and the lowest temperature (35.5 °C) was recorded in the boma-held rhinoceros after ~1 h of immobilisation.

Adverse effects recorded during immobilisation included muscle tremors and leg rigidity. Leg paddling occurred during lateral recumbency. Occasional ear twitching was recorded during immobilisation of most animals. One adult rhinoceros (Figure 1, individual # 2) that was immobilised in right lateral recumbency for 2.5 h developed lameness in the dependent (right) front leg, which healed after 3 days in a boma.

## Ethical considerations

The rhinoceroses included in the study were immobilised by the Wildlife Veterinary Unit in Zimbabwe for ongoing management purposes. Blood sampling was performed by experienced veterinarians with minimal discomfort for the animals because samples were collected when the rhinoceroses were immobilised. Physiological data and blood sample collection were part of the monitoring conducted during immobilisation. This contributes to *The principle of the 3Rs* through *reduction* - because no animals were immobilised solely for research purposes and to *refinement* - because identification of physiological alterations during immobilisation is the basis to enable establishment of methods for improvement. The study was conducted in accordance with international ethical guidelines.

## Discussion

Black rhinoceroses immobilised with the opioid-based drug combinations and the doses used in the present study developed marked hypoxaemia, which also has been reported during immobilisation with other drug combinations in both black and white rhinoceroses (Bush *et al*. 2004; Hattingh *et al*. 1994; Radcliffe *et al*. 2014; Wenger *et al*. 2007). In Zimbabwe, α_2_-adrenoceptor agonists are commonly included in drug combinations used for rhinoceros immobilisation to improve muscle relaxation. Despite nalorphine administration for partial reversal of the opioid effect, all but two rhinoceroses (92%) developed hypoxaemia, and most animals remained hypoxaemic throughout immobilisation. We also showed that body position had an effect on arterial oxygenation in immobilised black rhinoceroses; arterial oxygenation was higher during sternal than lateral recumbency.

The cause of the hypoxaemia and hypercapnia that developed is multifactorial. In white rhinoceroses, the suggested reasons for hypoxaemia are ventilation–perfusion (V_A_/Q) mismatch induced by recumbency and hypoventilation (increased PaCO_2_) because of opioid-induced respiratory depression (Bush *et al*. 2004; Hattingh *et al*. 1994; Heard *et al*. 1992). Intercostal muscle rigidity caused by opioids may prevent normal respiratory efforts, thus contributing to hypoventilation, as described in elephants and white rhinoceroses (Buss *et al*. 2015; Haw *et al*. 2014; Heard, Jacobson & Brock 1986). In addition, hypoventilation can be caused by upper-airway obstruction, for example, if the dependent nostril is occluded by the ground during lateral recumbency. The major contributors to hypoxaemia, as suggested by increased mean values of P(A-a)O_2_ in the present study, were intrapulmonary factors, such as V_A_/Q mismatch and shunt. Hypoventilation and reduced P_I_O_2_ (altitude, low barometric pressure) also contributed to hypoxaemia, but to a lesser extent than intrapulmonary factors. However, in some individuals, hypoventilation was the major underlying cause of hypoxaemia. Calculation of shunt fraction was not possible because mixed venous blood samples from the pulmonary artery could not be collected in the field. During anaesthesia and recumbency of large domestic species, such as horses, it has been shown that right-to-left vascular shunt, and not V_A_/Q mismatch, is the major contributor to hypoxaemia (Nyman & Hedenstierna 1989). Compression atelectasis because of abdominal organs pushing onto the diaphragm, has been suggested as a probable reason for the development of shunt and hypoxaemia (Nyman *et al*. 1990).

In the present study, the lower arterial oxygenation measured in black rhinoceroses during lateral compared to sternal recumbency is in line with previous reports in black rhinoceroses, white rhinoceroses and horses (Gleed & Dobson 1988; Radcliffe *et al*. 2014; Wenger *et al*. 2007). In Zimbabwe, immobilised rhinoceroses have routinely been positioned in sternal recumbency because this is associated with the least risk of complications. The only rhinoceros in the present study that developed forelimb lameness post-immobilisation had to remain in lateral recumbency for 2.5 h, because repositioning was not possible with the limited number of field personnel present. In horses, the risk for post-anaesthetic myopathy increases with duration of anaesthesia, and in animals placed in lateral recumbency, the triceps muscle is compressed (Johnston *et al*. 2004; Lindsay *et al*. 1985). Because prevention of hypoxaemia is important in reducing the occurrence of post-anaesthetic myopathy in horses (Friend 1981), it is likely that the same measure would be beneficial for rhinoceroses, in addition to minimising the duration of recumbency and optimising positioning.

The well-known respiratory depressant effects induced by the potent opioids used for immobilisation of rhinoceroses are mediated via µ-receptors. With the aim to alleviate these effects, opioid κ-agonist/µ-antagonists or partial agonists are sometimes included in the drug protocol. Kock *et al*. (1995) reported that the use of nalorphine in immobilised black rhinoceroses clinically improved respiratory rate and depth and haemoglobin oxygen saturation measured by pulse oximetry. In contrast, blood gas analysis showed that the black rhinoceroses in the present study remained hypoxaemic and hypercapnic despite administration of nalorphine. Likewise, arterial blood gases in white rhinoceroses demonstrated that severe hypoxaemia persisted despite nalorphine administration (Bush *et al*. 2004). Similarly, addition of the mixed opioid agonist–antagonist butorphanol did not prevent hypoxaemia or hypercapnia during immobilisation of white rhinoceroses (Boardman *et al*. 2014; Haw *et al*. 2015; Miller *et al*. 2013; Wenger *et al*. 2007). However, a single i.v. butorphanol dose (15 mg per mg etorphine) in combination with tracheal oxygen insufflation (30 L/min) fully corrected opioid-induced hypoxaemia and reduced hypercapnia in laterally recumbent subadult male white rhinoceroses during boma immobilisation, whereas oxygen alone did not correct hypoxaemia in the same study (Haw *et al*. 2014). Partial reversal of the opioid effects is promising, but so far, the evaluated drugs and doses have not been fully efficient for the prevention or treatment of hypoxaemia without the addition of oxygen. Further study is needed to develop effective and simple protocols for the prevention of hypoxaemia and hypercapnia in free-ranging rhinoceroses. Intranasal oxygen administration (5 L/min – 10 L/min) to both black and white rhinoceroses markedly improved arterial oxygenation in subadults and calves, irrespective of their body position (Fahlman 2008). In contrast, when intranasal oxygen (15 L/min) was administered to two severely hypoxaemic adult white rhinoceroses in lateral recumbency, the arterial oxygenation increased but remained within the hypoxaemic range (PaO_2_ 56 mmHg and 67 mmHg, respectively) (Fahlman 2008). Similarly, although tracheal oxygen insufflation at 30 L/min to subadult white rhinoceroses in lateral recumbency increased arterial oxygenation, animals remained hypoxaemic (Haw *et al*. 2015), whereas with the addition of butorphanol hypoxaemia was fully corrected (Haw *et al*. 2014). Oxygen supplementation is strongly recommended during immobilisation of wildlife, but needs further study in many wildlife species. The efficacy of oxygen therapy may be influenced by multiple factors, such as the oxygen flow rate and the degree of V_A_/Q mismatch and shunt (Fahlman 2014).

In the black rhinoceroses in the present study, hypoxaemia was detected by blood gas analysis, whereas pulse oximetry was unreliable because it generally overestimated the haemoglobin oxygen saturation. Similarly, inconsistent pulse oximetry values have been reported in various wildlife species, including white rhinoceroses (Fahlman *et al*. 2010; Haymerle, Knauer & Walzer 2016; Mich *et al*. 2008). Notably, the accuracy of pulse oximetry can vary between different pulse oximeters and probe sites. The readings require adequate perfusion at the probe site, and can be disturbed by light movement or reduced peripheral blood flow (Matthews, Hartke & Allen 2003). When monitoring immobilised rhinoceroses, it is essential to be aware of the limitations of pulse oximetry. Albeit simple to operate, pulse oximeters may not detect hypoxaemia or the efficacy of oxygen therapy. Thus, decisions on interventions should not be based solely on pulse oximetry.

The ‘gold standard’ for scientific evaluation of arterial oxygenation is blood gas analysis, which provides a measured value for PaO_2_ and a calculated value of SaO_2_. However, portable blood gas analysers calculate SaO_2_ with an algorithm based on human haemoglobin characteristics (e.g. p50 = 26 mmHg). Recently, two methods to adapt the human haemoglobin–oxygen dissociation algorithm to the blood of white rhinoceroses (p50 = 20 mmHg) and to determine the accuracy of pulse oximetry were presented (Haymerle *et al*. 2016). The study showed that the SaO_2_ values that were calculated using the species-adapted algorithms were ~15% higher than the values calculated by the blood gas analyser (Haymerle *et al*. 2016). Also, the oxygen saturation values recorded by pulse oximetry were much lower than the calculated SaO_2_ values. Therefore, this shows that species-specific studies are needed to improve the understanding of common monitoring equipment and to ensure correct interpretation of measurements.

In various wildlife species, thiafentanil alone or in drug combinations commonly result in recumbency in less than 4 min (Citino *et al*. 2001; Grobler *et al*. 2001) or a dose-dependent induction time (Janssen *et al*. 1993; Stanley *et al*. 1988), but arterial blood gases have seldom been reported (Smith *et al*. 2006). Because darting of black rhinoceroses in Zimbabwe often must take place in risky terrain, a fast induction is crucial and can possibly be improved further. The rapid induction induced by the thiafentanil doses used in two black rhinoceroses was comparable to etorphine. However, to ensure quick access to monitor and stabilise the animal after recumbency during the presented study, only two free-ranging black rhinoceroses were suitable for testing of thiafentanil. These two animals were among the rhinoceroses with the lowest oxygen tensions and with marked hypercapnia. Thus, further study on the use of thiafentanil and its physiological effects is needed.

Even though acid–base status is important for evaluation of capture and immobilisation, lactate concentrations are only occasionally reported in African wildlife. In the present study the highest lactate concentrations and the lowest pH values were measured in free-ranging rhinoceroses, in blood samples collected and analysed within 20 min of helicopter darting. The increased lactate concentrations were most likely caused by anaerobic metabolism because of intense physical exertion during the procedure of helicopter darting, which indicates an exercise-induced lactic acidaemia.

In some individuals, the low pH reflected a mixed respiratory and metabolic acidaemia, when elevated PaCO_2_ and lactate levels contributed to the decrease in pH. A pH < 7.2, as recorded in two rhinoceroses in the present study, can adversely affect cardiac contractility and lead to arrhythmias (DiBartola 2006). Capture myopathy has been reported in black rhinoceroses, but with no description of a presumably underlying lactate elevation (Basson & Hofmeyr 1975). Intense forced exercise during the induction should be avoided to minimise stress and the development of lactic acidaemia when capturing rhinoceroses. Death because of cardiac arrest during immobilisation in the wild has been reported in a black rhinoceros with severe acidosis (venous pH 7.16) (Stegmann *et al*. 2001). On the other hand, Adams *et al*. (2005) reported successful resuscitation of a captive black rhinoceros that developed respiratory arrest after profound cardiopulmonary depression, which was attributed to drug overdose. The resuscitation included endotracheal intubation followed by manual intermittent positive pressure ventilation with 100% oxygen and intravenous administration of diprenorphine.

### Limitations of the study

Data collection was carried out opportunistically during immobilisation of rhinoceroses for various management procedures. The study could have been strengthened if data had been collected at standardised time points, but this was not possible during the present field work.

## Conclusion

Black rhinoceroses are at high risk of developing hypoxaemia, hypercapnia and acidaemia during immobilisation with the drug combinations and the capture method reported here. Arterial oxygenation was higher in sternal compared to lateral recumbency. Pulse oximetry was unreliable in the detection of hypoxaemia compared to blood gas analysis. Hypoxaemia should be anticipated when immobilising rhinoceroses and preventive measures should be taken routinely. Positioning in sternal recumbency and routine use of oxygen supplementation are recommended in the management of immobilised rhinoceroses as measures to improve arterial oxygenation. Further studies to improve arterial oxygenation are recommended to increase the safety for immobilised rhinoceroses.
